# Detection of Hepatocyte Growth Factor (HGF) Ligand-c-MET Receptor Activation in Formalin-Fixed Paraffin Embedded Specimens by a Novel Proximity Assay

**DOI:** 10.1371/journal.pone.0015932

**Published:** 2011-01-21

**Authors:** Rajiv Dua, Jianhuan Zhang, Gordon Parry, Elicia Penuel

**Affiliations:** Research and Development, Oncology, Monogram Biosciences Inc., South San Francisco, California, United States of America; Health Canada, Canada

## Abstract

Aberrant activation of membrane receptors frequently occurs in human carcinomas. Detection of phosphorylated receptors is commonly used as an indicator of receptor activation in formalin-fixed paraffin embedded (FFPE) tumor specimens. FFPE is a standard method of specimen preparation used in the histological analysis of solid tumors. Due to variability in FFPE preparations and the labile nature of protein phosphorylation, measurements of phospho-proteins are unreliable and create ambiguities in clinical interpretation. Here, we describe an alternative, novel approach to measure receptor activation by detecting and quantifying ligand-receptor complexes in FFPE specimens. We used hepatocyte growth factor (HGF)-c-MET as our model ligand-receptor system. HGF is the only known ligand of the c-MET tyrosine kinase receptor and HGF binding triggers c-MET phosphorylation. Novel antibody proximity-based assays were developed and used to detect and quantify total c-MET, total HGF, and HGF-c-MET ligand-receptor interactions in FFPE cell line and tumor tissue. In glioma cells, autocrine activation of c-MET by HGF-c-MET increased basal levels of c-MET phosphorylation at tyrosine (Tyr) 1003. Furthermore, HGF-c-MET activation in glioma cell lines was verified by Surface Protein-Protein Interaction by Crosslinking ELISA (SPPICE) assay in corresponding soluble cell lysates. Finally, we profiled levels of

c-MET, HGF, and HGF-c-MET complexes in FFPE specimens of human Non-Small Cell Lung Cancer (NSCLC), Gastric Cancer, Head and Neck Squamous Cell, and Head and Neck Non-Squamous Cell carcinomas. This report describes a novel approach for the detection and quantification of ligand-receptor interactions that can be widely applied to measure receptor activation in FFPE preclinical models and archived FFPE human tissue specimens.

## Introduction

Arguably, the most significant opportunity to improve response rates for targeted therapeutics in solid tumors resides in the ability to accurately match patient specific molecular alterations to drugs that antagonize those alterations. This is the goal of personalized medicine for oncology. In that context, measuring receptor activation signatures constitutes an integral part of an overall diagnostic strategy aimed at identifying potentially responsive patients, stratifying patients for clinical trials and monitoring therapeutic responses to a given drug. Post-translational modifications such as phosphorylation either at the level of the receptor or downstream proteins, are likely to be better indicators of signal pathway activation and thus drug susceptibility, than mere quantification of receptor levels. In a clinical setting, formalin-fixation followed by paraffin embedding (FFPE) is the most common format of tissue preservation used by pathologists. This format maintains antigenicity and ensures excellent cellular morphology for diagnosis and immunohistochemistry (IHC) applications. However, detection of phosphoproteins in FFPE specimens is not robust in clinical settings, especially in clinical surgical tissue samples. One reason may be due to rapid dephosphorylation during ischemic stress after surgery [1], [2]. During ischemia, opportunistic phosphatases in the cell are activated and dephosphorylate proteins. It has been shown that when tissues are not fixed immediately the ability to detect phosphoproteins is lost within 60 minutes after biopsy [1]. Consequently, there is an urgent need to develop alternative methods to measure receptor activation. Although not all of the intricate details are known, there are a series of key steps leading to receptor activation and subsequent signaling of cell growth and proliferation. These key steps, ligand-receptor binding, receptor dimerization, and receptor transphosphorylation resulting in the production of substrate and adaptor protein binding sites, can be measured as potential indicators of receptor activation. One can also measure total receptor expression by IHC or FISH, or total ligand levels by either IHC or ELISA, however, these are not direct indicators of receptor activation.

Receptor tyrosine kinases (RTK) play essential functions in regulating important cellular processes [3]. Aberrant activation of RTK's resulting from either by mutation, gene amplification, or overexpression is significantly associated with many human cancers [4]. Consequently, RTK's are important therapeutic targets and a number of RTK-directed drugs have received regulatory approval for treatment of human cancers. c-MET is a disulfide-linked α/β heterodimeric cell surface tyrosine kinase receptor. Hepatocyte growth factor (HGF; also known as scatter factor) is the only known c-MET ligand [5]. Binding of HGF to c-MET triggers c-MET receptor activation via autophosphorylation of the cytoplasmic domain and recruitment of adaptor proteins that potentiates cell signaling [5]. Aberrant HGF/c-MET signaling via ligand dependent (both paracrine and autocrine) and ligand independent mechanisms is well documented in several human cancers [4], [5] and multiple therapeutic agents targeting this pathway are in clinical development [4]–[6]. c-MET phosphorylation has been reported in Non-Small Cell Lung (NSCLC) carcinoma, Head and Neck Squamous Cell Carcinoma (HNSCC), and other carcinomas [7]–[9]. Like most receptor kinases, improved methods for reliable measurement of c-MET receptor activation are needed.

In this report, we describe the development of assays to detect and quantify ligand-receptor complexes as surrogate measure of receptor activation. Using an antibody proximity-based technology (VeraTag), we describe a novel approach for the detection and quantification of the HGF-c-MET ligand-receptor complex in FFPE specimens including cell lines and human carcinoma tissues. To our knowledge, this is the first report describing detection and quantification of ligand-receptor complexes in the FFPE format. Additionally, we also describe the development of assays to detect and quantify total c-MET and HGF levels in FFPE specimens. Our approach demonstrates the potential of antibody proximity assays to provide reliable measurements of aberrant receptor activation in FFPE specimens.

## Materials and Methods

H441, H226, A549, H2170, MCF7, H661, Ln229, U87MG, Ln18, U138, U118, and H596 cell lines were obtained from ATCC (American Type culture collection, Manassas, VA) and maintained in DMEM/F12 (50∶50) containing 10% fetal bovine serum (Invitrogen, Carlsbad, CA), 1% penicillin-streptomycin, 2 mM L-Glutamine (CellGro, Manassas, VA). Antibodies used in this study are as follows: Mouse anti-c-Met (clone 3D4) (Invitrogen, Carlsbad, CA), Rabbit anti-c-Met (CVD13) (Invitrogen, Carlsbad, CA), Anti-c-MET (clone SP44) (Spring Biosciences, Pleasanton, CA), Human HGF affinity chromatography purified Anti-human HGF (R&D systems, Minneapolis, MN), Anti-human HGF Ab-2 (clone SBF5) (Thermo Scientific, Fremont, CA), human HGF neutralizing antibody (R&D systems, Minneapolis, MN), Phospho c-Met (Tyr 1003) (13D11) (Cell signaling, Beverly, MA), Phospho c-Met (Tyr1234/1235) (D26) (Cell signaling, Beverly, MA), Phospho c-Met (Tyr1349) (Cell signaling, Beverly, MA), Rabbit monoclonal (DA1E) IgG (Cell signaling, Beverly, MA), Horseradish peroxidase (HRP) conjugated secondary antibodies (Pierce Biotechnology, Rockford, IL). Frozen or formalin-fixed paraffin embedded human tumor blocks were obtained from Asterand (Detroit, MI). Head and Neck tumors were macrodissected to remove non-tumor related content. Human Non-Small Cell lung carcinoma and human Gastric carcinoma tissues used in the study had greater than 70% tumor content. Cell lines were fixed in 10% neutral buffered formalin for 1 h at 4°C (Sigma, St. Louis, MO) and formalin-fixed paraffin embedded (FFPE) pellets were prepared by as described [10]. VeraTag reporter molecules were synthesized as described earlier [US Patent 7,105,308]. VeraTag reporter and biotin conjugated antibodies were prepared as before [10].

### c-MET assay for formalin-fixed paraffin embedded (FFPE) cell lines and human tumor tissues

Sections were deparaffinized/rehydrated in xylene (2×5 minutes), 100% ethanol (2×5 minutes), 70% ethanol (2×5 minutes), and deionized water (2×5 minutes). Antigen retrieval was performed with DIVA decloaker antigen retrieval buffer (Biocare medical, Concord, CA) in a decloaking chamber (Biocare medical, Concord, CA). After retrieval, slides were cooled for 1 h at room temperature, washed in deionized water, and blocked with sample blocking buffer (10% goat serum, 1.5% BSA, 1x PBS) for 1 h at room temperature. After removal of blocking buffer, sections were incubated at 4°C overnight with fluorescein-reporter conjugated and Biotin conjugated c-Met antibodies prepared in blocking buffer. Anti-c-Met (CVD13) (Invitrogen, Carlsbad, CA) was used for VeraTag Pro11 reporter conjugation; Anti-c-Met (clone 3D4) (Invitrogen, Carlsbad, CA) was used for biotin conjugation). Fluorescein-reporter reporter conjugated c-Met antibody: 0.5 µg/ml; biotin conjugated c-Met antibody: 1 µg/ml. Isotype controls were performed as above except primary antibodies were replaced with the following antibodies: fluorescein-reporter conjugated c-Met antibody (CVD13): 0.5 µg/ml; Biotin conjugated IgG2a (1 µg/ml). Antibody mixture was removed by aspiration, sections were washed sequentially with buffer 1 (0.25% TX-100, 1x PBS) and buffer 2 (1x PBS). Samples were incubated for 1 h at room temperature with streptavidin-conjugated methylene blue (2.5 µg/ml) prepared in 1x PBS. Slides were washed with buffer 1 (0.25% TX-100, 1x PBS) (1×5 minutes) followed by 3 changes of deionized water. An illumination buffer (IB) (0.002x PBS) containing fluorescein (0.003pmol/L) and two CE internal markers was added on top of the section and samples were illuminated at 4°C for 1 h with in-house LED array that mediated photo-activated cleavage of fluorescein-reporter molecules. After illumination, buffer containing released reporters was collected, and run on a capillary gel electrophoresis system (ABI3100) (Applied Biosystems) under 6 kv for 50 s injection conditions for separation and detection of VeraTag reporters. Quantification of VeraTag reporter peaks was done using customized VeraTag quantitative software that normalized the peak area to the internal standard fluorescein, resulting in relative peak area (RPA) that is proportional to the concentration of the detected analyte. Tumor section area was measured using image J as described before [10]. The final value of VeraTag fluorescein-reporter was calculated by normalizing with tumor area (TA) and volume of the illumination buffer (IB) using formula RPA*IB/TA as described [10]–[12].

### HGF assay for FFPE cell lines and human tumor tissues

Antibody proximity assays were performed as described above except anti-c-Met antibodies were replaced with anti-human HGF antibodies. Following antibodies were used for the assay: Anti-human HGF (R&D systems, Minneapolis, MN) was used for VeraTag reporter conjugation, Anti-human HGF (clone SBF5) (Thermo Scientific, Fremont, CA) was used for biotin conjugation. Pro11 fluorescein-reporter conjugated Anti-human HGF antibody: 0.5 µg/ml; biotin conjugated Anti-human HGF antibody: 1 µg/ml. Isotype controls were performed as above except primary antibodies were replaced with the following antibodies: Pro11 fluorescein-reporter conjugated Anti-human HGF antibody (0.5 µg/ml); Biotin conjugated IgG1 (1 µg/ml).

### HGF/c-MET ligand-receptor assay for FFPE cell lines and human tumor tissues

Sections were deparaffinized/rehydrated, antigen retrieved, and blocked using a blocking buffer as described above. Samples were incubated overnight at 4°C with a mixture of unlabeled c-MET antibody (SP44) (1 µg/ml) and Pro11 fluorescein-reporter conjugated anti-human HGF antibody (0.5 µg/ml) prepared in blocking buffer. Antibody mixture was removed by aspiration, sections were washed sequentially with buffer 1 (0.25% TX-100, 1x PBS) and buffer 2 (1x PBS). Samples were incubated with biotin conjugated goat anti-rabbit antibody (1 µg/ml) for 1 h at room temperature. After this step, samples were washed sequentially with buffer 1 and 2, incubated with nutravidin-methylene blue (0.5 µg/ml), and illuminated as described above. Fluorescein-reporter peaks were normalized with tumor area as described previously [10]–[12]. Isotype control assay was performed as above except primary antibodies were replaced with the following antibodies: Rabbit monoclonal (DA1E) IgG (1 µg/ml); Pro11 fluorescein-reporter anti-human HGF antibody (0.5 µg/ml). Tumors exhibited signals at least 2-fold greater than the isotype assay control were scored positive for HGF/c-MET complexes.

### Immunoprecipitation and Western blotting

Cells were seeded in tissue culture dishes and incubated at 37°C until 60–70% confluence.

Cells were starved overnight in serum free medium and stimulated with various doses of HGF (PeproTechUS, Rockey Hill, NJ) at 37°C for 10 minutes. Cells were washed with cold PBS and lysed in buffer L (50 mM Tris-HCl (pH 7.5), 1% TX-100, 150 mM NaCl, 50 mM β-glycerophosphonate, 50 mM NaF, 1 mM Na_3_VO_4_ and a cocktail of protease inhibitors (Roche)). Lysates were incubated for 10 minutes on ice, centrifuged at 13,000 rpm at 4°C for 10 minutes, and clear supernatant was collected. Human tumor tissue lysates were prepared by first adding frozen tumors in liquid nitrogen and then grinding tumors using a pestle. Following sufficient grinding and mixing, cold buffer L was added, samples were incubated for 20 minutes, centrifuged at 13000 rpm, and clear supernatant was collected. Protein concentration was measured using bicinchoninic acid reagent (Pierce Biotechnology, Rockford, IL). Total cell lysates were run on SDS-PAGE and western blots were probed with various antibodies. Primary antibodies used for probing immunoblot are as follows: Anti-c-Met (clone 3D4) (Invitrogen, Carlsbad, CA), Anti-human HGF (clone SBF5) (Thermo Scientific, Fremont, CA), anti-β actin (Clone AC-74) (Sigma, St Louis, MO). Immunoprecipitations were carried out using standard procedures and captured on protein A/G beads (Santa Cruz Biotechnology, Santa Cruz, CA). We used 200 µg and 2 mg of total protein, respectively, for cell line and tumor lysates. Primary antibodies used for immunoprecipitation are as follows:

Anti-c-Met (clone 3D4) (Invitrogen, Carlsbad, CA), Anti-human HGF (clone A10)

(Enzo Life Sciences, Plymouth meeting, PA)

### Peptide ELISA

A series of 43 peptides that span the entire intracellular domain of c-MET (K955-S1390) were custom ordered (Elim biopharmaceuticals, Hayward, CA). Each peptide was 14–15 amino acids long and contained 5 amino-acid overlapping sequence with the adjacent peptide. Peptides were absorbed on the ELISA plates overnight at 4°C. Plates were blocked with 3% BSA in PBS containing 0.05% Tween 20 (PBSTw). Following 2 PBSTw washes, antibodies were added. Following 3 washes, bound antibody was detected with Horseradish peroxidase labeled secondary antibody.

### c-MET and HGF ELISA

Total c-MET ELISA assay was performed as per manufacturer's instructions using a kit (R&D systems, Minneapolis, MN). Total HGF ELISA assay was performed as per manufacturer's instructions using a kit (RayBiotech, Norcross, GA).

### Immunohistochemistry and H&E staining

Samples were deparaffinized/rehydrated and heat-retrieved as described above. Sections were blocked with blocking buffer (10% BSA, 1.5% BSA, 1 mg/ml human IgG, 1x PBS) for 1 h at room temperature and then incubated with either anti-c-Met (CVD13) (Invitrogen, Carlsbad, CA) (1∶400 dilution) or Anti-human HGF (clone SBF5) (Thermo Scientific, Fremont, CA) (1 µg/ml) for 1 h at room temperature. Sections were washed sequentially with wash buffer 1 (0.25% TX-100, 1x PBS) and wash buffer 2 (1x PBS) and incubated with secondary antibodies (Vector labs, Burlingame, CA). Visualization of the c-Met or HGF on the sections was done using an immunoperoxidase system (Vector labs, Burlingame, CA) as per manufacturer's instructions. Histologic score (H-score)  = %IHC3^+^×3 + %IHC2^+^×2 + %IHC1^+^×1 + %IHC0×0 was calculated as described [10]. H&E staining of the tissues were performed using standard procedures. Cell micrographs were taken using a digital image camera mounted on a leica microscope.

### Covalent cross-linking and Surface Protein-Protein Interaction by Cross-linking ELISA (SPPICE)

Covalent cross-linking using sulfo-EGS (Pierce Biotechnology, Rockford, IL) was done as described [13]. After cell lysis, extracted proteins were collected, and immunoprecipitated with Anti-human HGF antibody (clone A10) (Enzo Life Sciences, Plymouth meeting, PA). Eluted proteins were separated on SDS-PAGE using Tris-glycine Gels, transferred to nitrocellulose membrane, and immunoblotted with anti c-MET antibody (CVD13) (Invitrogen, Carlsbad, CA).

To detect and quantify complexes by SPPICE assay, cross-linked proteins were extracted after cell lysis and added to 96 well microtiter plate pre-coated with c-MET capture antibody (R&D systems, Minneapolis, MN). After overnight incubation at 4°C, liquid was removed, followed by 3 washes with wash buffer (0.05% Tween-20, 1xPBS). A biotin conjugated Anti-human HGF detection antibody (RayBiotech, Norcross, GA) was added, followed by 3 washes with 1xPBS. Detection of the complexes was performed by adding streptavidin labeled Horseradish peroxidase (RayBiotech, Norcross, GA). Plates were developed by adding substrate as per manufacturer's instructions (R&D systems, Minneapolis, MN). Isotype IgG control assay was performed as above except extracted protein samples were added to 96 well microplate pre-coated with mouse IgG antibody (Becton Dickenson, Franklin lakes, NJ). OD_450_ was calculated by subtracting isotype IgG background from the assay specific signals.

## Results

### Proximity-based assays for the detection and quantification of ligand, receptor, and ligand- receptor complexes

Proximity-based VeraTag assays are antibody-based detection methods to quantify proteins and protein complexes. This approach has been successfully applied to the measurement of HER2 receptor activation by detecting and quantifying HER2 and HER2 dimer levels in clinical FFPE specimens and soluble cell lysate formats [10]–[12], [14]–[15].

**Figure 1:**
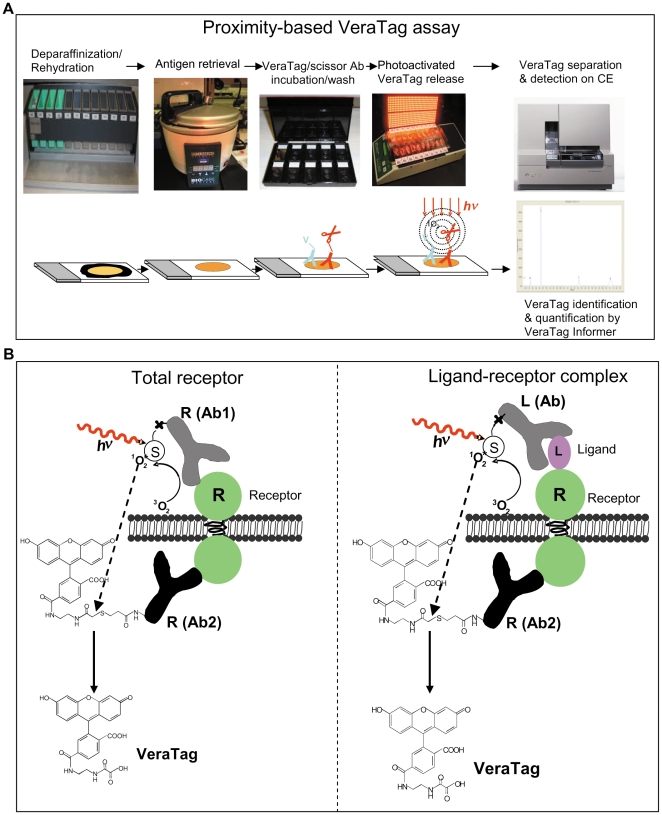
Antibody proximity VeraTag assay. A. Assay work-flow. B. Antibody proximity assay design for quantification of receptor and ligand-receptor complex.

A schematic of the FFPE assay work-flow is shown in Fig. 1A. Briefly, after deparaffinization and antigen retrieval, tissue sections are incubated with a mixture of fluorescein-reporter antibodies plus biotin-conjugated antibodies that recognize one or more target analytes. As a second step, a streptavidin (SA) conjugated photosenstizer (methylene blue; MB) is added which binds to the biotin-conjugated antibody. In a third step, the tissue section is illuminated with red light (670 nm) which triggers the SA-MB photosensitizer to convert dissolved O_2_ in the buffer to reactive singlet oxygen (^1^O_2_). This reactive state of the oxygen in turn cleaves thioether bonds linking the fluorescein- reporter to conjugated reporter antibodies that are bound in close proximity to the biotin-conjugated antibodies. The proximal selectivity of the ^1^O_2_ induced thioether cleavage is approximately 30 nm to 100 nm from the source of ^1^O_2_ [10]. In a final step, cleaved fluorescein reporters are collected from tissue sections, resolved by capillary electrophoresis, and quantified using customized VeraTag software.

Proximity assays can be configured to detect either receptor and ligand expression or ligand-receptor protein complexes by the appropriate selection of antibodies that target receptor and/or ligand. In proximity assays, receptor or ligand is detected and quantified by fluorescein-reporter and biotin-conjugated antibodies targeting different epitopes on the same protein (Fig. 1B). Conversely, to detect and quantify ligand-receptor complexes, the fluorescein-reporter conjugated antibody targets the receptor while the biotin-conjugated antibody targets the ligand (Fig. 1B) or vice versa.

### Detection and quantification of c-MET receptor in FFPE cell lines by antibody proximity assays

The expression of c-MET has been studied in many epithelial and mesenchymal cancers. In general, high expression of c-MET is associated with poorer prognosis. High c-MET expression in glioblastoma, breast cancer, gastric cancer, and ovarian cancer is associated with poor survival [16]–[18]. Despite the availability of semi-quantitative IHC assays for c-MET detection in FFPE tissues, a method that can quantify a continuous measurement of c-MET expression in tissues has not been developed.

We have developed a proximity assay for quantification of total c-MET expression in FFPE specimens. We procured multiple c-MET antibodies and assessed the specificity of each antibody in FFPE samples. For the initial assessment of antibody specificity, we used the lung cancer cell lines H1680 and H522 that express high and undetectable levels of c-MET, respectively. From these analyses, the c-MET (CVD13) rabbit polyclonal antibody and c-MET (clone 3D4) mouse monoclonal antibody were selected for further assay optimization.

Proximity assays were performed on a panel of FFPE specimens prepared Non-Small Cell Lung, Breast, and Glioma Cancer cell lines, using c-MET antibody pairs

**Figure 2:**
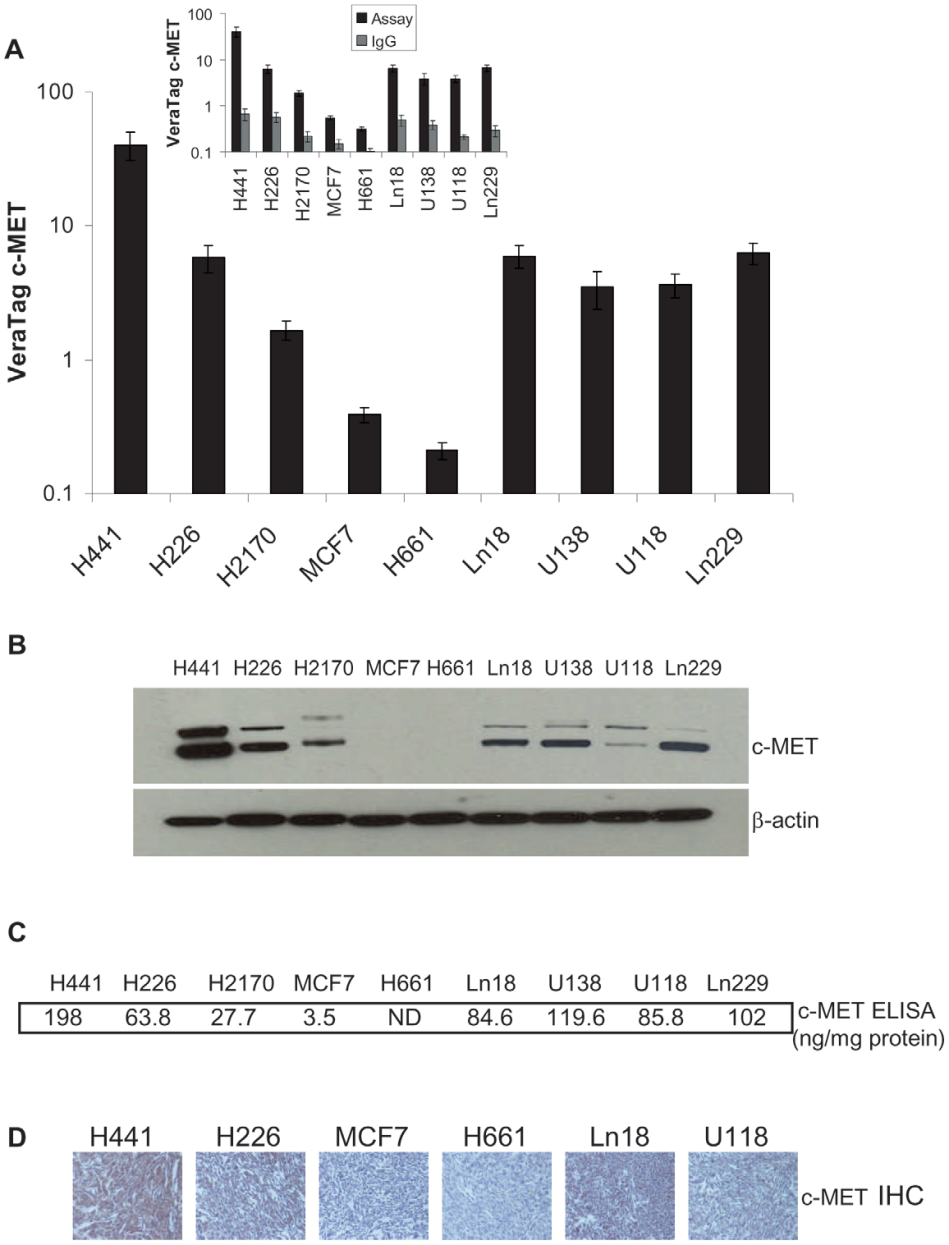
VeraTag FFPE c-MET quantification and correlation with Western blot, ELISA and IHC in cell lines. A. Quantification of c-MET expression in FFPE preparations of H441, H226, H2170, MCF7, Ln18, U138, U118, Ln229, U87, and H661 FFPE cell lines using the VeraTag assay. Background signals generated using an isotype control IgG (shown in inset) were subtracted from the c-MET assay signals. B. Western blot analysis of c-MET expression in H441, H226, H2170, MCF7, Ln18, U138, U118, Ln229, and H661 cell lysates. β-actin levels in the blot serves as a loading control. C. ELISA measurements of c-MET expression in H441, H226, H2170, MCF7, Ln18, U138, U118, Ln229, and H661 cell lysates. ND: Not detectable. D. IHC detection of c-MET expression in FFPE preparations of H441, H226, MCF7, Ln18, U118, and H661 cell lines.

CVD13 and clone 3D4 that were conjugated with either fluorescent reporters or biotin. Cell lines were selected based on their variable levels of c-MET protein expression: H441 express high levels of c-MET, H226 and H2170 express intermediate levels [19], [20], MCF7 cells express low, but detectable levels of c-MET [21] and H661 do not express c-MET [20]. We also used Ln18, U138, U118, and Ln229 glioma cell lines. Cell line pellets were prepared as FFPE specimens and analyzed for c-MET levels (Fig. 2A). The H441 cell line exhibited the highest signals while H226, Ln18, U138, U118, Ln229, and H2170 exhibited intermediate signals and the lowest c-MET were measured in MCF7 and H661 cells. Isotype control assays using antibody matched IgG generated signals that were less than 10% of the c-MET specific signals (Fig. 2A). Together, these data indicate that the dynamic range of c-MET proximity assays extends over a 2 log_10_ range.

In order to correlate FFPE proximity assay data with other independent biochemical methods, we prepared soluble cell lysates from the same panel of cell lines and conducted Western blot and ELISA analysis. FFPE proximity assay measurements (Fig. 2A) were generally concordant with Western blot (Fig. 2B) and ELISA (Fig. 2C) results. We also performed IHC on FFPE cell line pellets and as expected the c-MET staining pattern was highest in H441 cells and intermediate to low in the H226 and MCF7 cells (Fig. 2D). Taken together, the c-MET FFPE assay exhibited a broad range of specificity and data was consistent with independent assays including Western blot, ELISA and IHC.

**Figure 3:**
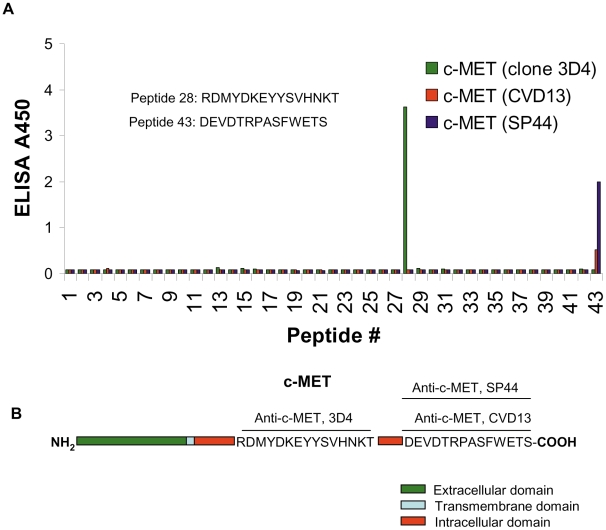
Epitope mapping of c-MET antibodies by peptide scanning. A. Peptide ELISA. A series of 43 overlapping peptides, each 14–15 amino-acid residues in length, spanning the complete intracellular domain of c-MET (K955-S1370), were evaluated to map the binding epitope of c-MET antibodies. Peptides were spotted on microtiter plates and ELISA assays were performed using standard protocols. The binding properties of peptide 28 and 43 localized the binding sites of c-MET antibody 3D4 and c-MET antibodies CVD13 and SP44, respectively. The sequence of peptide #28 and #43 are shown (inset). B. A schematic representing the c-MET receptor and binding sites for c-MET antibodies.

To further characterize the c-MET antibodies used in our proximity assay, we mapped their binding epitopes in an ELISA assay using a panel of overlapping peptides spanning the cytoplasmic domain of c-MET. This analysis indicated that the c-MET (clone 3D4) monoclonal antibody binds the epitope (^1227^RDMYDKEYYSVHNKT^1241^) in the C-terminus of c-MET, while the c-MET (CVD13) rabbit polyclonal binds the epitope (^1378^DEVDTRPASFWETS^1391^) at the extreme C-terminus (Fig. 3A, 3B). We also characterized a c-MET (clone SP44) rabbit monoclonal antibody in the c-MET FFPE assay. These data indicate that this rabbit monoclonal antibody (clone SP44) has c-MET specificity similar to the c-MET rabbit polyclonal (CVD13) antibody (data not shown). Characterization of this c-MET (clone SP44) antibody by epitope mapping analysis indicated that it binds to the epitope (^1378^DEVDTRPASFWETS^1391^) at the extreme C-terminus (Fig. 3A, 3B) similar to the c-MET (CVD13) rabbit polyclonal antibody and this enabled us to use this monoclonal antibody (clone SP44) as an alternative to the polyclonal c-MET (CVD13) antibody.

### Detection and quantification of c-MET receptor in human tumors by antibody proximity assay

**Figure 4:**
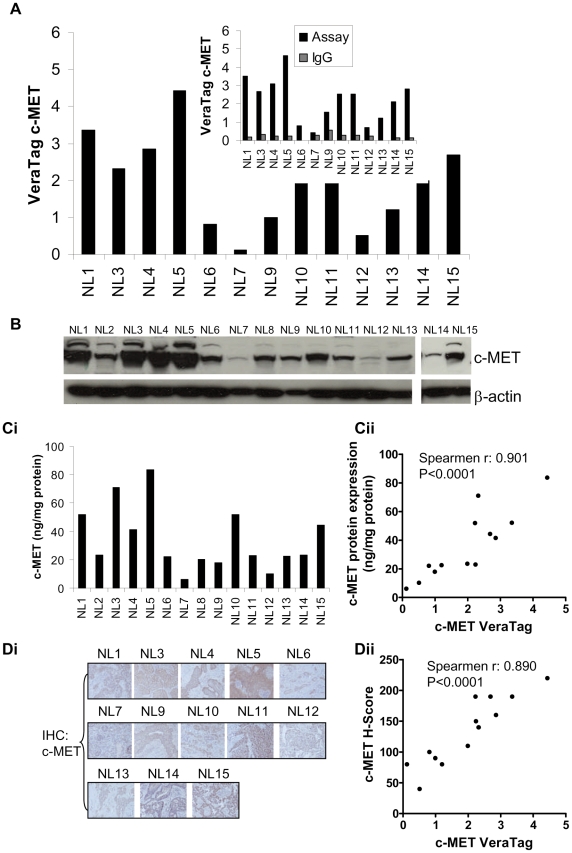
c-MET quantification using the VeraTag FFPE assay and correlation with Western blot, ELISA and IHC in human tumor specimens. A. Quantification of c-MET in NSCLC specimens using the VeraTag FFPE proximity assay. Isotype IgG antibody signal (inset) was subtracted from c-MET assay signals. B. Western blot analysis of c-MET in corresponding NSCLC tumor lysates. β-actin levels in the blot serves as a loading control. Ci. ELISA determinations of c-MET levels in corresponding NSCLC tumor lysates. Cii. Correlation of c-MET measurements in NSCLC specimens by proximity assay and ELISA. Di. c-MET detection in NSCLC by IHC. Dii. Correlation of c-MET measurements in NSCLC by FFPE VeraTag proximity assay and IHC. Histologic score (H-score)  =  %IHC3^+^×3 + %IHC2^+^×2 + %IHC1^+^×1 + %IHC0×0 was calculated as described [10]. Spearmen r and p values were calculated using graphpad prism software.

In order to characterize c-MET levels in human tumors, we obtained 15 human Non-Small Cell Lung (NSCLC) cancer specimens. Portions of each tumor were prepared as formalin-fixed paraffin embedded slides and soluble cell lysates (excluding sample NL2 and NL8 due to small tumor volumes). These NSCLC tumor samples displayed a ∼20-fold range of c-MET levels while isotype matched controls generated less than 10% of the c-MET specific signal (Fig. 4A). To compare our FFPE measurements with independent biochemical methods, we analyzed soluble lysates from the same panel of tumors using Western blot (Fig. 4B) and ELISA (Fig. 4Ci). Generally, c-MET FFPE measurements were concordant with Western blot and ELISA data. A two-tailed comparison of c-MET protein levels measured in FFPE specimens by proximity assay and by ELISA in soluble lysates was statistically significant (Spearmen r: 0.91; p<0.0001) (Fig. 4Cii). We also evaluated c-MET levels by IHC staining (Fig. 4Di) and the comparison of FFPE c-MET levels by IHC H-score and the proximity assay correlated with statistical significance (Spearmen r: 0.89; p<0.0001) (Fig. 4Dii). Taken together, our results demonstrate that the c-MET FFPE assay can detect and quantify c-MET receptor levels in FFPE tumor specimens, and these measurements correlate well with measurements obtained by conventional methods.

### Detection and quantification of hepatocyte growth factor (HGF) in cell lines by antibody proximity assays

**Figure 5:**
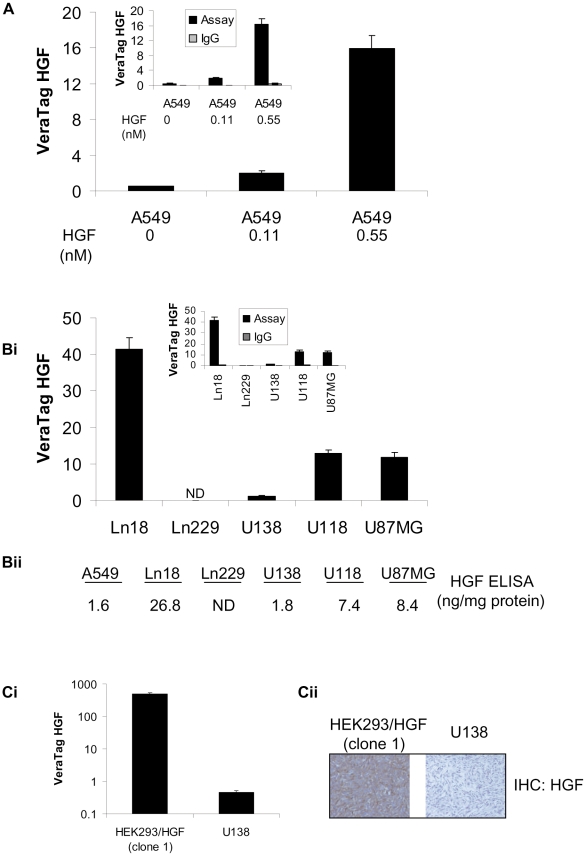
VeraTag FFPE quantification of HGF and correlation with Western blot, ELISA and IHC in cell lines. A. VeraTag FFPE quantification of HGF in unstimulated and HGF stimulated A549 cells. Isotype IgG control signals (inset) were subtracted from the HGF signals. Bi. VeraTag quantification of HGF in Ln18, Ln229, U138, U118, and U87MG glioma cell lines. Isotype IgG control signals (inset) were subtracted from HGF assay signals. ND: Not detectable. Bii. ELISA determinations of HGF in A549, Ln18, Ln229, U138, U118, and U87MG cell lysates. ND: Not detectable. Ci. VeraTag FFPE quantification of HGF in HEK293/HGF (clone 1) and U138 glioma cell lines. Cii. IHC detection of HGF in HEK293/HGF stable clone (clone 1) and U138 glioma cells.

Several studies have shown an association of serum or tumor associated HGF with the progression of various carcinomas [22], [23]. High levels of HGF in resected NSCLC are correlated with poor survival [23]. Despite the clinical significance of HGF overexpression, a quantitative assay that measures HGF in FFPE specimens has not been reported. To address this unmet need, we applied our antibody proximity technology to the development of a quantitative assay that measures HGF expression levels in FFPE tumor specimens. A large panel of HGF antibodies was screened for specificity using a stable cell line (HEK293/HGF (clone 1) that overexpress HGF and the HGF negative parental cell line (HEK 293). Using these cell lines, we identified a goat polyclonal anti-human HGF antibody and a mouse monoclonal anti-human HGF antibody (clone SBF5) that exhibited high specificity. The proximity assay was then optimized in two antibody format using A549 cells in the presence of increasing amounts of HGF (Fig. 5A). In agreement with a previous report [24], unstimulated A549 cells exhibited low HGF expression (Fig. 5A). HGF measurements using isotype antibody matched controls were less than 5% of the HGF specific assay signals (Fig. 5A).

Next, we quantified HGF in cell lines that exhibit autocrine HGF/c-MET signaling. We assessed endogenous level of HGF in FFPE pellets generated from a panel of glioma cell lines (Ln229, Ln18, U138, U118, and U87MG cells). U138, U118, and U87MG possess an HGF/c-MET autocrine loop and secrete HGF in the culture medium [23]. U118, Ln18, and U87MG exhibited ∼12–40 fold higher levels of HGF than U138 cell line (Fig. 5Bi), while Ln229 cells produced no detectable HGF signal. These measurements correlated well with the HGF ELISA measurements we obtained using the corresponding glioma cell lysates (Fig. 5Bii) and are in agreement with previous reports on endogenous HGF expression in HGF in U138 and U118 cells [25].

The dynamic range of the HGF proximity assay was defined as ∼3 log_10_ by comparing the assay signals of the HEK293/HGF cell line against the U138 cell line (Fig. 5Ci) and this is consistent with detection of HGF by IHC in corresponding cell lines (Fig. 5Cii).

### Detection and quantification of hepatocyte growth factor (HGF) in human tumors by antibody proximity assays

**Figure 6:**
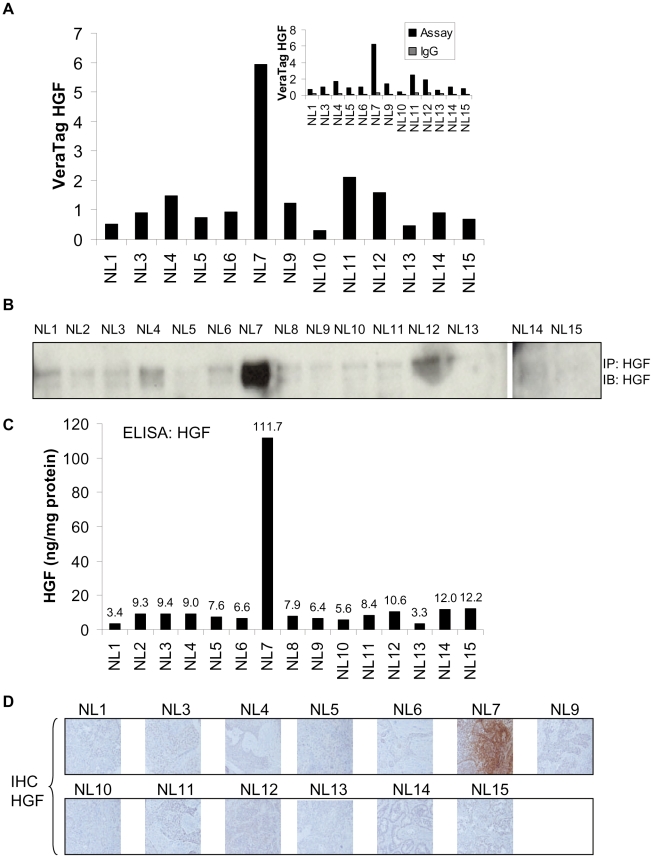
VeraTag FFPE quantification of HGF and correlation with Western blot, ELISA, and IHC in human tumor specimens. A. VeraTag FFPE quantification of HGF in NSCLC tumors. Isotype IgG control signals (inset) were subtracted from HGF signals. B. Immunoprecipitation/Western blot analysis of HGF in corresponding NSCLC tumor lysates. C. ELISA determinations of HGF in corresponding NSCLC specimens. D. IHC detection in NSCLC specimens.

We utilized the same NSCLC tumor panel that was used for the evaluation of the c-MET proximity assay to evaluate the HGF proximity assay (Fig. 6A). NL7 was the only sample that exhibited high HGF expression levels in this panel. Isotype antibody controls signals were consistently less than 20% of HGF antibody-specific signals (Fig. 6A). To compare HGF proximity assay measurements to independent biochemical methods, we performed Western blot and ELISA assays on the corresponding tissue lysates. HGF was immuno-precipitated from 2 mg of cellular protein and probed for HGF by Western blot analysis. Consistent with the HGF proximity assay, sample NL7 exhibited high levels of HGF compared to the other samples (Fig. 6B). ELISA analysis of the HGF levels in these samples was also consistent with both the proximity assay and Western blot measurements (Fig. 6C). Furthermore, the results of the HGF proximity assays, ELISA, and Western blot were all concordant with IHC staining (Fig. 6D).

### Detection and quantification of HGF/c-MET ligand-receptor interaction in FFPE cell line pellets by antibody proximity-based assay and correlation with c-MET (pY1003) phosphorylation

**Figure 7:**
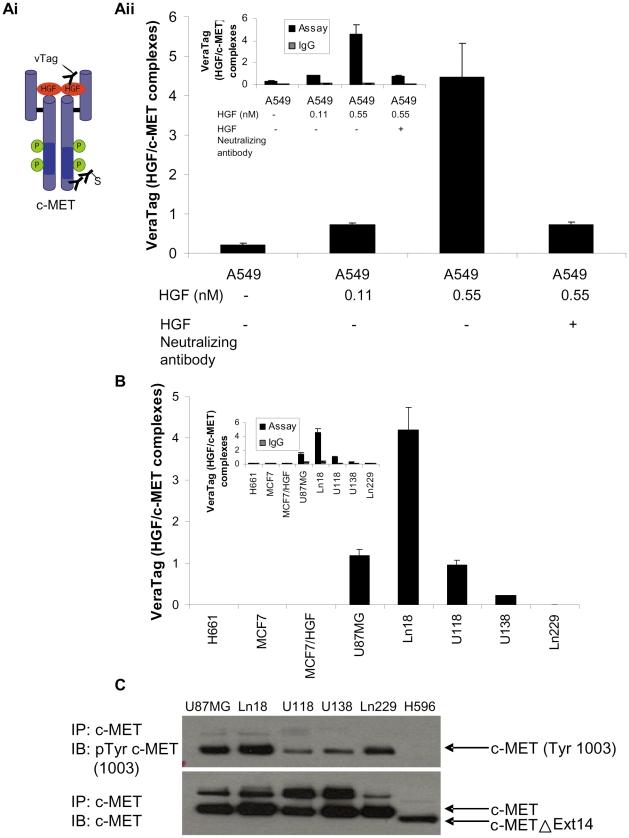
VeraTag FFPE quantification of HGF/c-MET ligand-receptor complexes and correlation with c-MET (Tyr1003) phosphorylation. Ai. Schematic of the HGF/c-MET VeraTag FFPE proximity assay. Aii. Quantification of HGF/c-MET levels in unstimulated and HGF stimulated A549 cells. Isotype IgG control signals (inset) were subtracted from the HGF/c-MET assay signals. B. VeraTag quantification of HGF/c-MET ligand receptor complexes in H661, MCF7, HGF stimulated MCF7, Ln18, Ln229, U138, U118, U87MG cell lines. C. Immunoprecipitation/Western blot analysis of c-MET (Tyr1003) phosphorylation and c-MET in Ln18, Ln229, U138, U118, and U87 MG glioma cells. The H596 cell line with exon 14 (L964-D1010) deletion was included as a negative control for c-MET (Tyr1003) phosphorylation.

We used anti c-MET and anti-HGF antibodies that worked well in the c-MET and HGF assays to develop a quantitative assay for detection of HGF/c-MET complexes in FFPE specimens. As illustrated in Fig. 7Ai, the assay is performed by (a) incubation of FFPE samples with a rabbit anti-MET antibody (clone SP44) together with a human anti-HGF antibody that is conjugated to a VeraTag reporter, (b) addition of biotin-conjugated goat anti-rabbit antibody which binds to the rabbit anti-MET (clone SP44) antibody, (c) addition of streptavidin-methylene blue, which serves as photosensitizer, and (d) illumination of the sample, which activates the photosensitizer and releases VeraTag reporters in close proximity (see Fig. 1A). This secondary antibody format enhances signal strength which in turn improves assay sensitivity and reproducibility (Wallweber J, Unpublished data). A similar format has been applied to the quantitative measurements of the truncated form (p95) of the HER2 receptor in breast cancer [11]. To demonstrate the ability of our new assay to detect the HGF/c-MET complex, we performed the assay on unstimulated and HGF stimulated A549 cells. As illustrated in Fig. 7Aii, we observed increases in the HGF/c-MET complex in the HGF stimulated A549 cells which were proportional to the dosage of HGF. To assess the extent of assay background resulting from non-specific antibody binding, replicate samples (adjacent sections) were incubated with the VeraTag labeled anti-HGF antibody and an isotype (IgG DA1E) in place of the anti-MET antibody. In this case, isotype control signals were less than 20% of the HGF/c-MET complex specific signals (Fig. 7Aii (inset)). Additionally, we could demonstrate a decrease in the HGF/c-MET complex signal in HGF treated A549 cells when HGF was pre-incubated with an HGF neutralizing antibody (clone 24612) (Fig. 7Aii). To rule out that this decrease in HGF/c-MET signal was not due to assay interference by neutralizing antibody binding, we verified that the addition of a 10-fold excess of HGF neutralizing antibody did not interfere with the assay (data not shown). Furthermore, we also observed a corresponding decrease in c-MET phosphorylation when HGF was pre-incubated with the HGF neutralizing antibody (data not shown).

Next, we evaluated whether the FFPE assay could be used to detect endogenous levels of HGF/c-MET complexes in glioma cell lines that activate c-MET signaling through the autocrine production of HGF [25]. As illustrated in Fig. 7B, the HGF/c-MET complex was detected in the Ln18, U138, U118, and U87MG cell lines but not in the Ln229 cells (Fig. 7B). This result is consistent with the HGF and c-MET data we present in Fig. 2A and Fig. 5Bi, respectively, i.e. Ln229 cells express intermediate levels of c-MET but not endogenous HGF. Once again, we verified that the signals from isotype control assays were less than 20% of the HGF/c-MET specific assay signals (Fig. 7B (inset)). Furthermore, there was no significant difference in the HGF/c-MET and isotype control signals for MCF7 and H661 cell lines which express very low or no c-MET and do not express endogenous HGF (Fig. 7B).

Next, we sought to correlate the presence of autocrine driven HGF/c-MET complexes in glioma cells with a direct indicator of c-MET activation. Upon ligand activation, c-MET is phosphorylated, most notably at amino acid positions Y1003, Y1234/1235, and Y1349. In addition, c-MET phosphorylation (pY1003) is a marker of HER1 inhibitor (gefitinib) resistance in NSCLC patients [26]. We measured c-MET pY1003 levels by immuno-precipitation in lysates prepared from the Ln18, Ln229, U118, U138, and U87MG glioma cell lines to compare with the HGF/c-MET levels we detected using the VeraTag FFPE assay format. The H596 cell line containing a deletion in c-MET exon 14 that removes amino acids L964 through D1010 [27] was used as a negative control for our c-MET (pY1003) immuno-precipitation studies. As illustrated in Fig. 7C, higher basal c-MET (pY1003) phosphorylation was detected in Ln18 and U87MG cells relative to U138 and U118 cells. This observation is consistent with the detection of higher levels of HGF/c-MET complex in Ln18 and U87MG cells relative to U138 and U118 cells using the VeraTag FFPE assay format (Fig. 7B). Interestingly, the Ln229 cell line that lack measurable levels of the HGF/c-MET complex in the FFPE assay (Fig. 7B) exhibited elevated levels of c-MET (pY1003) phosphoryaltion in the lysate immunoprecipitation assay (Fig. 7C).

### Detection of HGF/c-MET ligand-receptor complexes by Surface Protein-Protein Interaction by Cross-linking ELISA (SPPICE) assay

**Figure 8:**
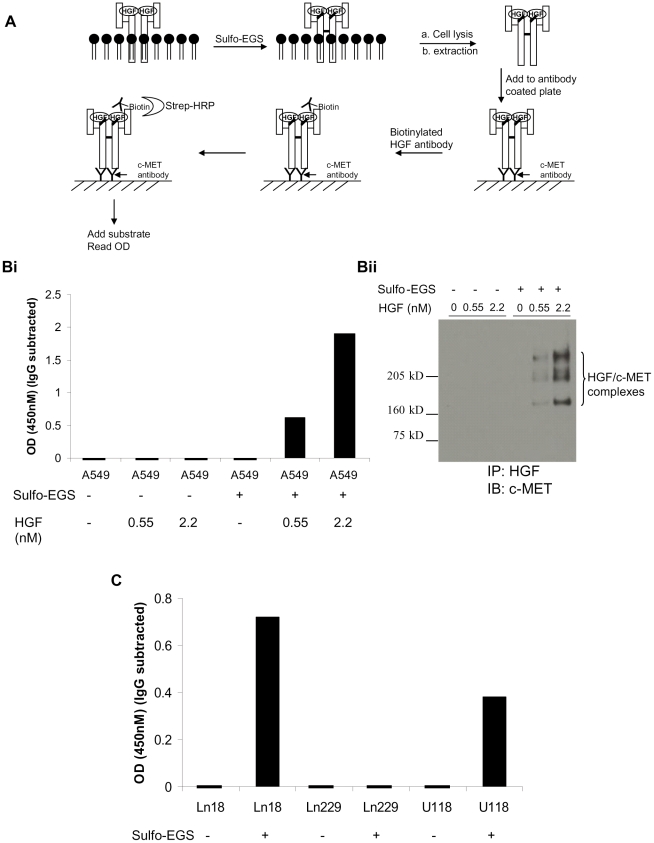
Measurement of the HGF/c-MET complex by Surface Protein-Protein Interaction by Cross-linking ELISA (SPPICE) assay. A. SPPICE schemetic. c-MET was chemically cross-linked to expgenous HGF using the membrane impermeable sulfo-EGS cross-linker. Membrane proteins were solublized and HGF/c-MET complexes were measured using the SPPICE procedure (Bi) or by immunoprecipitation with HGF antibodies followed by Western blotting with anti-c-MET antibodies (Bii). C. c-MET and endogenous HGF in glioma cells were cross-linked as described above and SPPICE was used to measure HGF/c-MET complexes.

We developed a novel biochemical method for detection of the HGF/c-MET complex to cross-validate the VeraTag HGF/c-MET assay. Chemical cross-linking of proteins is commonly used to demonstrate protein-protein interactions, therefore, we combined chemical crosslinking with ELISA based detection to develop Surface Protein-Protein Interaction by Cross-linking ELISA (SPPICE). A schematic of the HGF/c-MET SPPICE assay is depicted in Fig. 8A, and details of the assay are described in Materials and Methods. Briefly, cell cultures expressing surface c-MET receptors in the presence of either exogenous or endogenous HGF are treated with a membrane impermeable sulfo-EGS cross-linker. Soluble cross-linker proteins are subsequently extracted and the lysates are applied to 96 well microtiter plates pre-coated with c-MET antibody. A biotinylated HGF antibody is added and HGF/c-MET complexes are detected by further addition of streptavidin-HRP and colorimetric substrate. SPPICE was used to assess HGF/c-MET levels in unstimulated or HGF stimulated A549 cells. We detected HGF dose-dependent increases in the HGF/c-MET complex only when the cells were treated with the sulfo-EGS cross-linker (Fig. 8Bi). In the absence of the cross-linker treatment, the HGF/c-MET complex was not detected in either unstimulated and HGF stimulated A549 cells. The detection of HGF/c-MET ligand-receptor complexes by conventional SDS-PAGE and Western blot analysis further corroborated the SPPICE results (Fig. 8Bii). To assess endogenous levels of HGF/c-MET ligand-receptor complexes in glioma cells, we used SPPICE to characterize Ln18, Ln229, and U118 cell lysates. Consistent with the proximity FFPE assay (Fig. 7C), SPPICE detected HGF/c-MET complexes in Ln18 and U118 cells but not in Ln229 cells (Fig. 8C).

### Profiling c-MET, HGF, and HGF/c-MET ligand-receptor complexes in human NSCLC, Gastric, and Head and Neck tumors by antibody proximity assays

**Figure 9:**
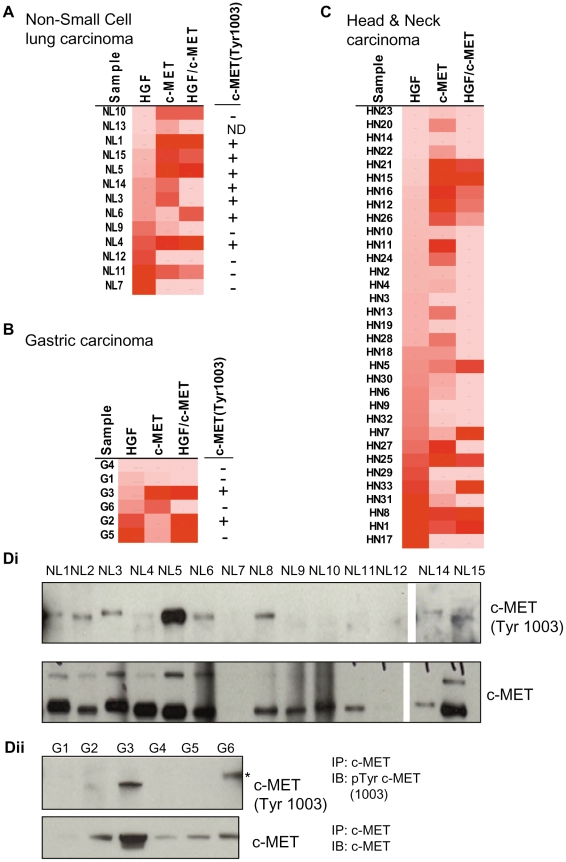
c-MET, HGF, and HGF/c-MET complexes in human carcinomas. A. NSCLC. B. Gastric carcinoma. C. Head and Neck carcinoma. c-MET, HGF, and HGF/c-MET assays were performed for all FFPE samples. Samples were rank ordered from low to high levels of HGF. The levels of c-MET receptor and HGF/c-MET were also tabulated. Red color-High ≥90^th^ percentile; Pink color-Low ≤10^th^ percentile. NSCLC and Gastric tumor exceeded 70% tumor content by pathologic analysis. In Head & Neck carcinoma samples, non-tumor tissue was removed by macrodissection. Di. c-MET was immunoprecipitated from NSCLC lysate samples and immunoblotted using c-MET (Tyr1003) or c-MET antibodies. Dii. c-MET was immunoprecipitated from gastric tumor lysates and immunoblotted using c-MET (Tyr1003) or c-MET antibodies. Samples that were c-MET phosphorylation (pY1003) positive (+) or c-MET (pY1003) negative (−) are summarized in Fig. A, B.

We measured HGF/c-MET ligand-receptor complexes in the same NSCLC carcinoma tumor panel that was used for individual c-MET and HGF measurements. c-MET, HGF, and HGF/c-MET levels were also measured in six gastric carcinomas and 33 Head and Neck (HN) carcinomas. The HN tumors included 17 squamous cell and 16 non-squamous cell carcinomas. Tumors were rank ordered based on increasing HGF levels and corresponding c-MET and HGF/c-MET complex measures are represented as a heat map in Figs. 9A, B, C. The HGF/c-MET complex was detected in 7 of 13 (54%) NSCLC specimens (NL1, NL4, NL5, NL6, NL11, NL15), 3 of 6 (50%) gastric tumors (G2, G3, G5), and 11of 33 (33%) HN tumors (HN1, HN5, HN7, HN8, HN12, HN15, HN16, HN21, HN25, HN26, HN33). Within HN group, HGF/c-MET was detected in 7 of 17 (40%) squamous cell carcinomas (HN1, HN5, HN7, HN8, HN21, HN25, HN26), and 4 of 16 (25%) non-squamous cell carcinomas (HN12, HN15, HN16, HN33). Tumors identified positive for HGF/c-MET ligand-receptor complexes exhibited signals that were at least 2-fold greater than the isotype assay control signals (data not shown). A combined analysis of NSCLC, gastric, and HN tumor measurements did not reveal significant correlations between HGF/c-MET levels and c-MET expression (pearson r  = 0.1782; p = 0.2063) or HGF expression (r = -0.021; p = 0.8794). It is important to note that HGF/c-MET measurements were not simply the products of the individual HGF and c-MET measurements (pearson r = 0.1164; p = 0.4112). We also compared our HGF/c-MET measurements in the proximity FFPE assay with the levels of c-MET (pY1003), c-MET (pY1234/1235), and c-MET (pY1349) phosphorylation detected in NSCLC and gastric tumors. Due to the low abundance of phosphorylated c-MET protein in tumor samples, c-MET was immunoprecipitated prior to immuno-detection by Western blot (Fig. 9Di, ii). c-MET (pY1003) phosphorylation was detected in 9 NSCLC tumor lysates (NL1, NL2, NL3, NL4, NL5, NL6, NL8, NL14, and NL15) and the HGF/c-MET was also detected in 5 of 6 corresponding FFPE sections by proximity FFPE assay (NL1, NL4, NL5, NL6, and NL15). Two tumors (NL2, NL8) were too small (<20mm^2^) to run in the FFPE format. c-MET (pY1003) phosphorylation was detected in only two gastric tumor lysates (G2, G3) and these same tumors were also positive for HGF/c-MET complexes by proximity FFPE assay. In one gastric tumor lysate (G6), a slightly higher molecular weight/slower migrating band that also reacted with the c-MET (pY1003), however, at the present time, the identity of this protein is uncertain (Fig. 9Dii). Our attempts to detect c-MET (pY1234/1235) and c-MET (pY1349) phosphorylation in NSCLC and gastric tumors were unsuccessful (data not shown) suggesting that phosphorylation at these sites might be labile than c-MET (pY1003).

## Discussion

The c-MET, HGF, and c-MET/HGF assays described in this report are based on the release of antibody conjugated fluorescein reporters by antibody conjugated molecular scissors that are bound in close proximity. This technology has been used to measure total HER2 receptor and HER2 homodimer levels in breast tumor FFPE specimens [10], [28] and several studies have demonstrated the clinical utility of these measurements [11], [12], [28]. In this report, we used the HGF/c-MET ligand-receptor interaction as a model for the development of a ligand-receptor proximity assay as a method to measure receptor activation in FFPE specimens.

We have generated assays to quantify the expression of c-MET, HGF, and HGF/c-MET ligand-receptor complexes in FFPE cell lines and human carcinomas and have cross-validated these measurements using independent biochemical methods. The expression of c-MET and HGF is associated with tumor progression and a number of clinical studies have implicated the prognostic and predictive value of c-MET and/or HGF measurements [16], [17], [18], [22], [23]. Based on the AQUA™ platform commercially available c-MET antibodies demonstrate lot to lot variation that result in poor reproducibility of c-MET measurements [29]. We screened a large panel of commercially available c-MET and HGF antibodies and optimized a pair of antibodies for use in proximity assays. The use of two antibodies that bind in close proximity undoubtedly contribute to the high specificity of detection of the assays described here.

To develop a HGF/c-MET ligand-receptor assay, we utilized the c-MET and HGF antibodies that worked well in our FFPE assays. We also incorporated a secondary antibody detection format that enhances assay signal strength and reproducibility [11]. In cell lines and tumors that were positive for HGF/c-MET, signals were more than 2 fold higher than the isotype IgG control signals. In addition, detection of endogenous levels of HGF/c-MET in glioma cells was consistent between proximity FFPE and SPPICE biochemical assays. c-MET phosphorylation was used to demonstrate that HGF/c-MET detection can serve as a surrogate measure of c-MET activation. In several glioma cells that exhibit activation of HGF/c-MET signaling, we detected high levels of c-MET (pY1003) phosphorylation that are likely modulated through a c-MET and HGF autocrine loop. In contrast, our measurements of HGF, c-MET, HGF/c-MET, and c-MET phosphorylation suggest that c-MET activation in Ln229 cells is not the result of autocrine signaling. At present, we do not understand the mechanism of constitutive c-MET phosphorylation in this cell line, but it is conceivable that signaling is mediated through an alternative co-receptor or as yet unidentified c-MET activating mutations.

c-MET activation has recently been shown to play important roles in HN carcinoma [2] via a paracrine mechanism in which HGF is secreted by surrounding stromal cells [30]. Consistent with this observation, we detected c-MET phosphorylation in NSCLC and gastric tumors that also contained measurable levels of HGF/c-MET complex. In NSCLC, Zucali et al [1] reported a significant correlation between c-MET (pY1003) phosphorylation and resistance to gefitinib treatment and rapid disease progression. A similar association between gefitinib resistance and HGF induced c-MET activation has also been reported for lung adenocarcinomas containing activating EGFR mutations [26]. Although, we detected c-MET (pY1003) phosphorylation in human tumors, our attempts to measure c-MET (pY1234/1235) and c-MET (pY1349) phosphorylation were unsuccessful. It is possible that pY1003 c-MET phosphorylation is inherently more stable than c-MET (py1234/1235) phosphorylation. Alternatively, the kinetics of site-specific phosphorylation may be different. A study of tyrosine phosphorylation of c-MET in a renal carcinoma cell line suggested that c-MET (pY1349) phosphorylation occurs 5 minutes after HGF treatment and persists for 120 minutes while c-MET (pY1234/1235) phosphorylation occurs 30 minutes after HGF treatment and is subject to rapid turnover [31].

Strategies to identify relevant biomarkers in parallel with development of targeted therapeutics is essential for the successful selection of appropriate clinical trial subjects and eventual treatment of potentially responsive patients. A number of approaches for HGF/c-MET biomarker identification in a clinical setting have been proposed including the measurement of plasma HGF and shed c-MET [32]. Patients treated with a therapeutic antibody against HGF (AMG102), exhibited increased plasma HGF levels but no significant changes in shed c-MET [32]. In a phase I of c-MET MetMab (Anti-MET therapeutic antibody), a single patient that experienced a complete response exhibited a diagnostic profile consistent with autocrine biology [33]. In another study, c-MET phosphoryaltion was reduced in the post-operative tumor biopsies of patients treated with a c-MET kinase inhibitor (ARQ197) [34]. The VeraTag proximity assays described here present a unique opportunity to provide quantitative measurements of c-MET receptor activation that may contribute to the development of antagonistic c-MET therapeutics.
